# Fast genetic mapping of complex traits in *C. elegans* using millions of individuals in bulk

**DOI:** 10.1038/s41467-019-10636-9

**Published:** 2019-06-18

**Authors:** Alejandro Burga, Eyal Ben-David, Tzitziki Lemus Vergara, James Boocock, Leonid Kruglyak

**Affiliations:** 1 Department of Human Genetics, Department of Biological Chemistry, and Howard Hughes Medical Institute, University of California, Los Angeles, Los Angeles, CA 90095 USA; 2 Present Address: Institute of Molecular Biotechnology of the Austrian Academy of Sciences (IMBA), Vienna, Austria

**Keywords:** Genetic linkage study, Quantitative trait

## Abstract

Genetic studies of complex traits in animals have been hindered by the need to generate, maintain, and phenotype large panels of recombinant lines. We developed a new method, *C. elegans* eXtreme Quantitative Trait Locus (*ce*X-QTL) mapping, that overcomes this obstacle via bulk selection on millions of unique recombinant individuals. We use *ce*X-QTL to map a drug resistance locus with high resolution. We also map differences in gene expression in live worms and discovered that mutations in the co-chaperone *sti-1* upregulate the transcription of HSP-90. Lastly, we use *ce*X-QTL to map loci that influence fitness genome-wide confirming previously reported causal variants and uncovering new fitness loci. *ce*X-QTL is fast, powerful and cost-effective, and will accelerate the study of complex traits in animals.

## Abstract (web summary)

QTL mapping in multicellular animals usually requires generation, maintenance, and phenotyping large panels of recombinant individuals. Here, the authors develop *ce*X-QTL mapping, a method for fast genetic mapping via bulk selection on millions of unique recombinant *C. elegans* individuals.

## Introduction

Most heritable traits have a complex genetic architecture. Quantitative trait locus (QTL) mapping has been pivotal in identifying loci underlying complex traits of medical, agricultural, and evolutionary importance^1–6^. However, genetic studies of complex traits remain challenging, especially in multicellular organisms. QTL mapping usually relies on generating large panels of cross progeny that must be individually genotyped and phenotyped. The construction and maintenance of such panels is lengthy, laborious, and costly, limiting the size of most studies and their statistical power to confidently detect and narrow the genomic position of loci.

**Fig. 1:**
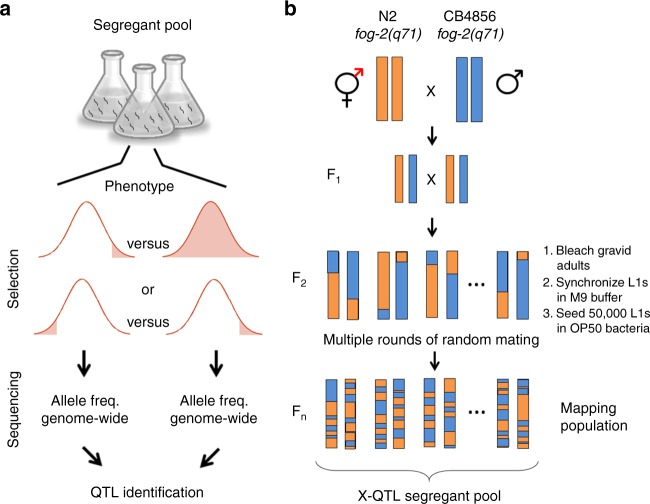
Implementing eXtreme quantitative trait loci (X-QTL) in *C. elegans*. **a** The principle underlying X-QTL. A large population of recombinant individuals is selected based on a trait of interest. Two types of experiments are possible. Either one tail of the distribution is selected and contrasted with the overall population or two tails of the distribution are selected and contrasted with each other. Each group is then genotyped in bulk, and differences in allele frequency between these groups identify QTLs. **b** To generate millions of unique recombinant individuals in *C. elegans*, we used parental strains carrying the *fog-2(q71)* allele, which abolishes selfing. The resulting population can only reproduce by outcrossing. *C. elegans* experiences only one crossover event in each bivalent per meiosis, leading to an effective rate of half a recombination per chromosome. To increase the total number of recombination events and the resolution of *ce*X-QTL, we grew the segregant pool for multiple non-overlapping generations. In every generation, eggs were isolated by hypochlorite treatment from fully gravid adults. L1 larvae were synchronized by overnight starvation in M9 buffer and seeded on nematode growth medium plates (50,000 individuals)

An alternative to traditional QTL mapping is bulked segregant analysis (BSA)^7^. In the original BSA approach, cross progeny are still generated and phenotyped individually, but then individuals that fall into the tails of the phenotypic distribution are pooled for genotyping in bulk, and allele frequencies in the pools are compared to identify QTLs^8,9^. Building on the foundation of BSA and similar approaches^10^, our laboratory developed eXtreme QTL (X-QTL) mapping in the budding yeast *S. cerevisiae*^11^. In X-QTL, generation of cross progeny, genotyping, and phenotyping are all carried out in bulk, enabling the use of extremely large populations of segregants (>10^6^ individuals), with correspondingly high statistical power and mapping resolution (Fig. 1a). Using X-QTL, we have successfully resolved the genetic architecture of numerous complex traits in yeast, such as natural variation in resistance to chemicals, mitochondrial function, and gene expression^11–14^. However, we lack an equivalent powerful, fast, and cost-effective method that can scale up to millions of individuals in animals.

Here we extend the X-QTL approach to the nematode *C. elegans*. We show that *C. elegans* X-QTL (*ce*X-QTL) can be used to quickly map loci underlying differences in drug and stress resistance, gene expression, and fitness.

## Results

### Development of *C. elegans* X-QTL

X-QTL requires the generation of a large population of genetically unique segregants. Self-fertilization, the primary mode of reproduction of *C. elegans*, poses a challenge to implementing X-QTL because selfing individuals do not contribute to the genetic diversity of the segregant pool. To adapt the life cycle of *C. elegans* to X-QTL, we genetically abolished hermaphroditism using a *fog-2(q71)* mutation that “feminizes” hermaphrodites by eliminating their sperm production^15^ (Fig. 1b). For simplicity, we will refer to these worms as females because they can only reproduce by crossing with males.

To generate the X-QTL segregant pool, we used two highly divergent *C. elegans* parental strains: the N2 reference strain (Bristol, UK) and the wild isolate CB4856 (Hawaii, USA). We constructed the CB4856 X-QTL parental strain by introgressing the *fog-2(q71)* allele into the Hawaiian background (Supplementary Fig. 1). We then crossed N2 *fog-2(q71)* females to CB4856 *fog-2(q71)* males and propagated a population of 50,000 segregants for 12 non-overlapping generations (Fig. 1b). We propagated the segregant population for multiple generations to increase the total number of recombination events per chromosome in the pool and, consequently, the mapping resolution^9,16^. Extensive simulations showed that this population size and number of generations provide sufficient genome-wide mapping power to detect loci explaining as little as 0.5% of phenotypic variance (“Methods” and Supplementary Figs. 2–4). A population of 50,000 is easy to maintain in the laboratory, and it can be quickly expanded to millions of individuals in a single generation because each female lays hundreds of eggs.

### Mapping natural genetic variation in drug resistance

Avermectins are a family of drugs widely used to treat parasitic worm infections and to fight insect pests. Thus resistance to avermectins is a major health and agricultural problem^17^. We previously mapped a locus contributing to natural variation in Abamectin (Avermectin B1) resistance by studying the effect of this drug on locomotor activity in *C. elegans*^18^. Abamectin paralyzes N2 at a faster rate than CB4856. To find the variant underlying this phenotypic difference, we originally performed QTL mapping using a large panel of 210 recombinant inbred advanced intercross lines (RIAILs). In each of these lines, sensitivity to Abamectin was determined by studying the frequency of body bends in liquid^18^.

**Fig. 2:**
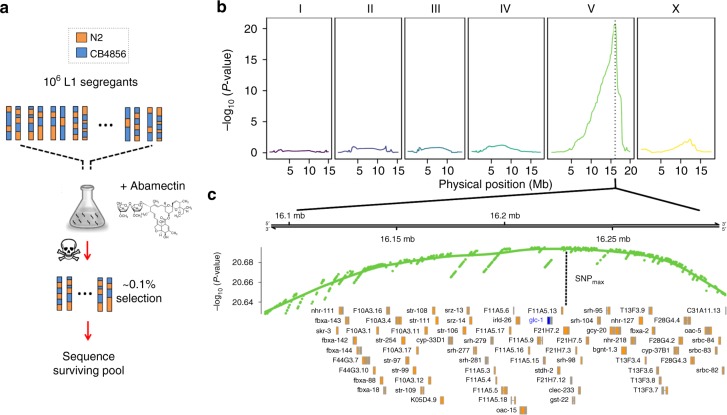
Mapping of natural resistance to Abamectin. **a** To map natural variation in Abamectin resistance using *C. elegans* eXtreme quantitative trait loci (*ce*X-QTL), we treated four million F_12_ L1 recombinant larvae from a N2 × CB4856 cross with 0.2 µg/mL of Abamectin in M9 for 1 min. Abamectin (Avermectin B1) is a natural fermenting product of the bacteria *Streptomyces avermitilis* and acts as a nerve poison by binding invertebrate-specific glutamate-gated chloride channels. We then sequenced the surviving population (~0.1%) and a control population that was exposed only to dimethyl sulfoxide (vector) and calculated genome-wide allele frequencies. **b** *ce*X-QTL identified a highly significant QTL peak on the right arm of Chr. V (*p* = 5.3 × 10^−22^). Dashed vertical line denotes the 95% confidence interval (CI): 16,115,957–16,276,907 Mb. **c** Focusing on the CI region, genes in orange. Dashed vertical line shows the single-nucleotide variant with the lowest *p* value in the region, which is located 3.7 kb away from *glc-1* (blue), the alpha-subunit of the glutamate-gated chloride channel. Natural variation in *glc-1* confers resistance to Abamectin^18^

In addition to affecting locomotor activity in adult worms, high doses of Abamectin can be lethal^19^. We reasoned that resistance to Abamectin could be mapped in bulk by exposing a large number of N2 × CB4856 recombinant L1 larvae to a lethal dose of Abamectin and sequencing the surviving segregant pool. We treated four million F_12_ L1 recombinant larvae with 0.2 µg/mL of Abamectin in M9; only ~0.1% of the population survived this treatment (Fig. 2a). We extracted genomic DNA from the surviving population and from a control population that was exposed to dimethyl sulfoxide (DMSO) alone. Finally, we estimated genome-wide allele frequency of 110,176 single-nucleotide variants (SNVs) using Illumina short-read sequencing and mapped QTLs by implementing a statistical framework previously developed for BSA^20^.

*ce*X-QTL revealed a highly significant locus contributing to Abamectin resistance on Chr. V (Fig. 2b, 95% confidence interval 16,115,957–16,276,907 Mb; *p* = 5.3 × 10^−22^), in the same region identified in the large RIAIL panel. The confidence interval obtained using *ce*X-QTL was smaller than the one obtained using the 210 RIAIL panel (224 kb for RIAIL panel^18^ and 160 kb for *ce*X-QTL). However, we cannot exclude the possibility that differences in the phenotypic assays could be at least partly responsible for the increased mapping resolution. The SNV with the most significant *p* value was located only 3.7 kb away from the gene *glc-1* (Fig. 2c). We previously showed that *glc-1*, which encodes the alpha subunit of a glutamate-gated chloride channel, is the causal gene underlying the QTL^18,21^.

In addition to drug resistance, *ce*X-QTL can also be used to map variation in any trait that can be selected for in bulk. For instance, we also subjected a population of 1.5 million L1 segregants to oxidative stress (0.5 mM H_2_O_2_) and uncovered 2 significant QTLs (Chr. II; *p* = 1.60 × 10^−5^ and Chr. IV; *p* = 3.07 × 10^−18^; Supplementary Fig. 5). These results illustrate the power of X-QTL in *C. elegans* to quickly guide the mapping of QTL segregating in the wild in a single and fast experiment using a large mapping population.

### Coupling X-QTL and worm sorting

**Fig. 3:**
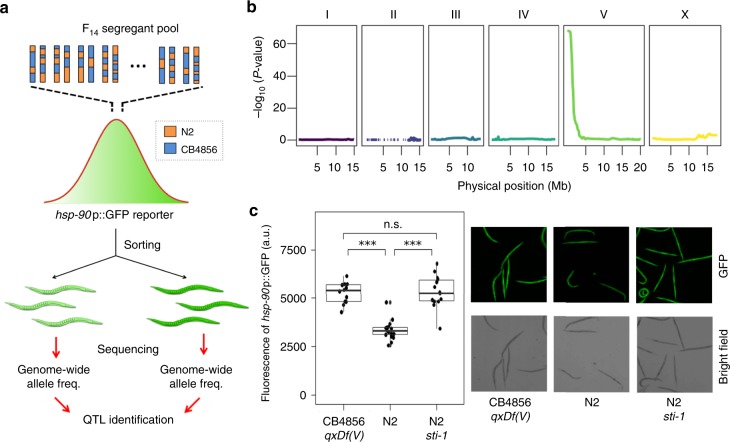
*C. elegans* eXtreme quantitative trait loci (*ce*X-QTL) identifies a regulator of *hsp-90* transcription. **a** To map variants underlying gene expression differences between isolates, we coupled *ce*X-QTL to a large particle biosorter. We crossed a transcriptional *hsp-90*::GFP reporter into our parental N2 *fog-2(q71)* and CB4856 *fog-2(q71) ce*X-QTL strains and propagated a segregant population for 14 generations. We then measured the green fluorescent protein (GFP) fluorescence of ~60,000 F_14_ recombinant young adults and selected ~2000 individuals from the two tails of the distribution (“High” and “Low” GFP). We sequenced both populations and calculated genome-wide allele frequencies. **b** *ce*X-QTL mapping identified a highly significant QTL peak in the left arm of Chr. V (*p* = 9.56 × 10^−69^). **c** A loss-of-function allele in the co-chaperone, *sti-1*, is sufficient to recapitulate the transcriptional upregulation of *hsp-90* observed in the parental CB4856 strain carrying *qxDf(V)*, a 267-kb de novo deletion. Quantification of *hsp-90*::GFP reporter expression (left) and representative images (right). Boxplots; center line is the median and the box limits are the upper and lower quartiles. All data points are shown as black circles. *Hsp-90* reporter expression was significantly higher in CB4856 *qxDf(V)* compared to N2 (1.58-fold upregulation, *p* = 7.9 × 10^−6^) and in N2 *sti-1(ok3354)* mutants compared to N2 (1.60-fold upregulation, *p* = 8.1 × 10^−6^). Hsp-90 reporter expression was not significantly different between CB4856 *qxDf(V)* and N2 *sti-1(ok3354)* (*p* = 0.6). *p* values were calculated using two-sided Wilcoxon rank-sum test. ***: *p* < 0.0005

Our laboratory previously combined X-QTL and fluorescence activated cell sorting to study the genetics of protein abundance in yeast^14^. To develop an analogous approach in *C. elegans*, we coupled *ce*X-QTL to the Union Biometrica large-particle Biosorter, an instrument capable of viably sorting whole live worms. To demonstrate the power of this approach, we studied the transcriptional regulation of *C. elegans hsp-90 (daf-21)*, a highly conserved chaperone that is constitutively expressed throughout *C. elegans* development^22^. To test whether *hsp-90* expression levels vary between isolates, we introgressed a single-copy *hsp-90p::GFP* transcriptional reporter from the N2 background into CB4856. We observed higher expression of this reporter throughout all developmental stages in the CB4856 background (2.6-fold upregulation in embryos; *p* = 1.0 × 10^−4^ and 1.7-fold upregulation in adults; *p* = 2.2 × 10^−6^; Supplementary Fig. 6). To map QTLs underlying this difference, we crossed the *hsp-90* reporter into our parental N2 *fog-2(q71)* and CB4856 *fog-2(q71)* X-QTL strains and propagated a segregant population for 14 non-overlapping generations. We then measured the green fluorescent protein (GFP) fluorescence of ~60,000 F_14_ recombinant young adults and selected ~2000 individuals from each of the two tails of the distribution (“High” and “Low“ GFP) (Fig. 3a, Supplementary Fig. 7). *ce*X-QTL analysis revealed a single highly significant locus on the left arm of Chr. V (*p* = 9.56 × 10^−69^, Fig. 3b). The “High” GFP population was enriched for CB4856 alleles in the QTL region, as expected based on the parental phenotypes (Supplementary Fig. 7).

Close inspection of the locus on Chr. V revealed a large 267-kb deletion in the CB4856 *hsp-90p*::GFP strain (Chr. V:565,773–833,171 Mb) (Supplementary Fig. 8). This large deletion is not present in the original CB4856 parental strain, indicating that it was most likely acquired de novo during the introgression of the *hsp-90* transcriptional reporter into CB4856 and is not a natural polymorphism. Very little is known about the transcriptional regulation of *hsp-90*^23^ other than the role of *hsf-1*, the master regulator of the heat shock response. Therefore, we decided to further investigate the underlying causal mutation to gain insights into the regulation of this essential chaperone.

The Chr. V deletion encompassed 117 genes (Supplementary Data 1). We reasoned that the causal gene should be constitutively expressed during all developmental stages because the *hsp-90p::GFP* transcriptional reporter is upregulated in all tissues throughout the life of the worm. We leveraged gene expression data from the *C. elegans* modEncode project^24^ and filtered our candidate list by keeping only those genes that were constitutively expressed during embryonic and larval development. This analysis reduced our list from 117 to 20 genes. We screened these 20 genes using RNAi on the parental *hsp-90p::GFP* N2 strain. RNAi silencing of only one gene, *sti-1*, caused upregulation of the *hsp-90* reporter (Supplementary Data 1). *C. elegans sti-1* is the ortholog of mammalian Hop and yeast Sti1, a co-chaperone that binds the chaperones Hsp90 and Hsp70^22,25^. Hop/Sti1 inhibits the ATPase activity of Hsp90 by acting as a non-competitive inhibitor and stabilizing the Hsp90 open conformation^26^. To confirm this finding, we studied a strain carrying the *sti-1(ok3354) allele*, a 336-bp deletion removing the last 90 amino acids of the protein. In agreement with our RNAi screen results, *sti-1* mutants showed upregulation of the *hsp-90p::GFP* reporter to a level indistinguishable from the CB4856 introgression strain (Fig. 3c). Together, these experiments indicate that *sti-1* loss of function causes transcriptional upregulation of *hsp-90*. Overall, our results illustrate how *ce*X-QTL in conjunction with a large particle biosorter can be used to study the genetics of gene expression in *C. elegans*. This strategy can be further extended to study natural variation in any trait amenable to sorting in living worms, including reporters of stress–response and lifespan^27^, mitochondrial activity^28^, maternal provisioning^29^, diet^30^, metabolism^31^, and neuronal activity^32^.

### *ce*X-QTL identifies loci influencing competitive fitness

Fitness, the measure of the reproductive success of an individual, is a complex genetic trait of fundamental importance to evolution. Without variation in fitness, adaptation cannot occur. However, genome-wide mapping of genetic variants influencing fitness remains challenging and has largely been limited to microorganisms^33^. Throughout our experiments, we noticed that several genomic regions showed marked changes in allele frequencies that were shared by both our control segregant populations and those under selection. Although such baseline changes in allele frequencies do not hamper *ce*X-QTL mapping (Supplementary Fig. 9), we reasoned that they could reflect selective forces. To gain further insights into the origin of these allele frequency deviations, we sampled and sequenced earlier generations of the segregant pool, which were stored in frozen stocks.

**Fig. 4:**
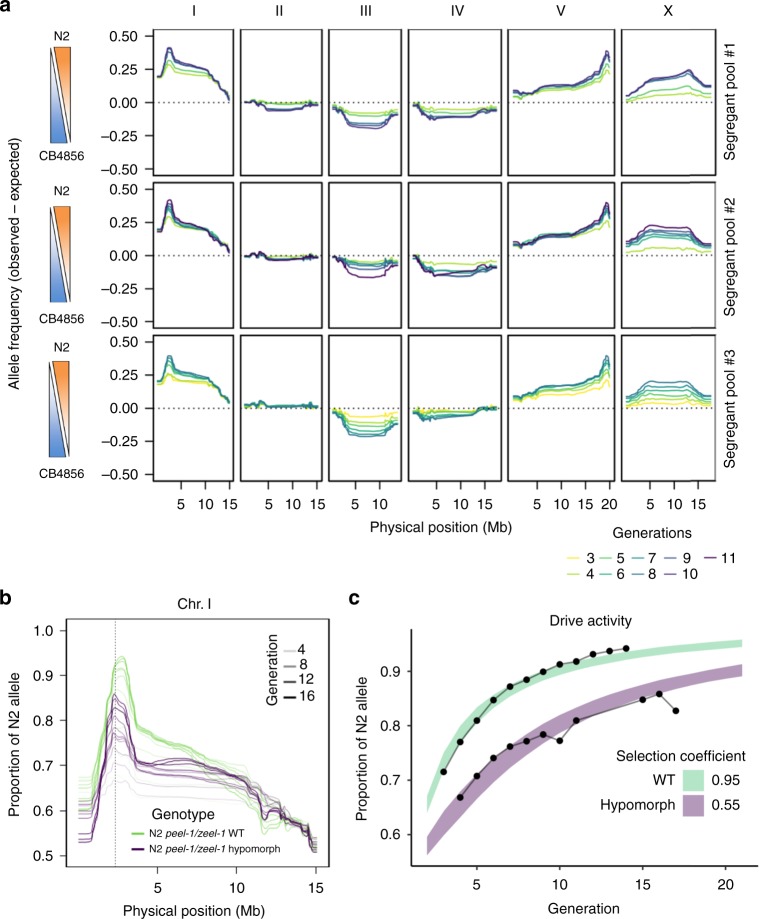
Temporal dynamics of *C. elegans* eXtreme quantitative trait loci segregant pools reveal loci influencing competitive fitness. **a** Changes in allele frequencies across multiple generations (ranging from F_3_ to F_11_) in three biological repeats of an N2 × CB4856 cross. Allele frequencies correspond to the difference between the observed and the expected values. Each of these pools originates from an independent cross of parental strains performed months apart. **b** Driving activity of the *peel-1/zeel-1* selfish element in an N2 × CB4856 segregant population across multiple generations. N2 *peel-1/zeel-1* WT allele (green) and hypomorphic N2 *peel-1(ttTi12715)/zeel-1* allele (purple). The dashed vertical line denotes the location of the *peel-1/zeel-1* selfish element on Chr. I. **c** Estimation of selection coefficients from the observed changes in allele frequencies at the *peel-1/zeel-1* locus using simulations. The fitness of the poisoned homozygous individual lacking the selfish element is 1 − s, where “*s*” is the coefficient of selection. For a fully penetrant toxin-antidote element like *peel-1/zeel-1 allele, s* = 1. The estimated “*s*” for both wild-type (WT) and hypomorph alleles were 0.95 and 0.55, respectively. Bands are the 95% confidence intervals (CI) of simulations for WT (green) and hypomorph (purple)

We observed highly consistent deviations from the expected allele frequencies over the course of multiple generations (Fig. 4a; experiments can be explored in depth using our cexQTLview app—https://github.com/eyalbenda/cexQTLview). To exclude the possibility that these changes were the result of genetic drift, we independently generated and propagated two additional N2 × CB4856 segregant pools. Changes in allele frequencies were highly reproducible across biological repeats (Fig. 4a), suggesting that numerous genomic regions were most likely conferring fitness advantages.

One of the most extreme shifts in allele frequencies was observed on the left arm of Chr. I, where the *peel-1/zeel-1* selfish element is located^34,35^.The SNV with the largest deviation in allele frequency was located 40–100 kb away from the ~40 kb highly divergent structural variant spanning the *peel-1/zeel-1* element. This selfish element is composed of two tightly linked genes: *peel-1*, a sperm-delivered toxin, and *zeel-1*, its zygotically expressed antidote. The N2 strain carries both genes, whereas CB4856 lacks them. In crosses between heterozygous individuals, only the progeny that inherits at least one copy of the element survives, resulting in 25% embryonic lethality. We observed a progressive increase in the frequency of the N2 *peel-1/zeel-1* haplotype in the segregant pool, in agreement with its selfish “gene drive” activity (Fig. 4a, b).

We wondered whether the observed changes in allele frequency of loci across generations could be used to quantify the relative strength of selection. To test this idea, we studied *peel-1(ttTi12715)*, a hypomorphic allele of the *peel-1* toxin carrying a transposon insertion^36^, and compared its fixation dynamics to those of the N2 wild-type (WT) allele. As expected, the drive activity of *peel-1(ttTi12715)* was not abolished, but it was reduced compared to that of the WT allele (Fig. 4b). To quantify this effect, we compared the observed results with simulations, allowing us to estimate the selection coefficient (*s*). We found that selection was much weaker for the hypomorph, illustrating the sensitivity of our assay (*s*_wt_ = 0.95, *s*_hypomorph_ = 0.55; Fig. 4c). Thus our multigenerational *ce*X-QTL segregant approach is not only effective in mapping QTLs but it can also be used to quantify the relative strength of selection on loci influencing fitness.

A large peak on the left arm of Chr. X includes the neuropeptide receptor *npr-1*^37^. N2 carries a gain-of-function dominant mutation in *npr-1* that increases fecundity^38^. Replacement of the N2 *npr-1* allele with its CB4856 counterpart abolished the fitness peak on the left arm of Chr. X, thus confirming that *npr-1* was driving this signal (Supplementary Fig. 10a). In addition to known variants that contribute to fitness, we also uncovered several novel loci. For example, we found that almost the entirety of CB4856 Chr. III was selected over its N2 counterpart. This is particularly surprising, because, in contrast to CB4856, the N2 strain has been selected for growth in the laboratory for over 50 years. We hypothesized that *plg-1* could underlie the strong selection in favor of CB4856 Chr. III due to male–male competition. *plg-1* encodes a mucin-like gene that is required in *C. elegans* to form a copulatory plug. In N2, this gene is disrupted by a transposon insertion, while it is functional in CB4856 and many other wild isolates^39^. However, reintroducing a functional *plg-1* allele into the N2 background did not affect the selection in favor of the CB4856 Chr. III, indicating that other unknown variants underlie this difference in fitness (Supplementary Fig. 10a).

To further evaluate the reproducibility of the fitness peaks detected in our segregant pools, we studied a CB4856 *fog-2(kah89)* knock-in strain generated using CRISPR/Cas9 (Supplementary Fig. 10). We crossed N2 *fog-2(q71)* females to CB4856 *fog-2(kah89)* males and propagated the segregant pool for 17 generations. Notably, with the exception of a peak on the right end of Chr. V and a secondary peak in Chr. X, we could reproduce all the major fitness effect loci that we observed using the CB4856 *fog-2(q71)* introgression strain (Fig. 4a and Supplementary Fig. 10b). The signal on the right end of Chr. V is most likely driven by a de novo mutation in close linkage with the *fog-2(q71)* introgression. These results show that direct gene editing by CRISPR/Cas9 is an effective method to generate parental *ce*X-QTL strains and that it avoids effects that can arise from de novo mutations introduced during allele introgression.

**Fig. 5:**
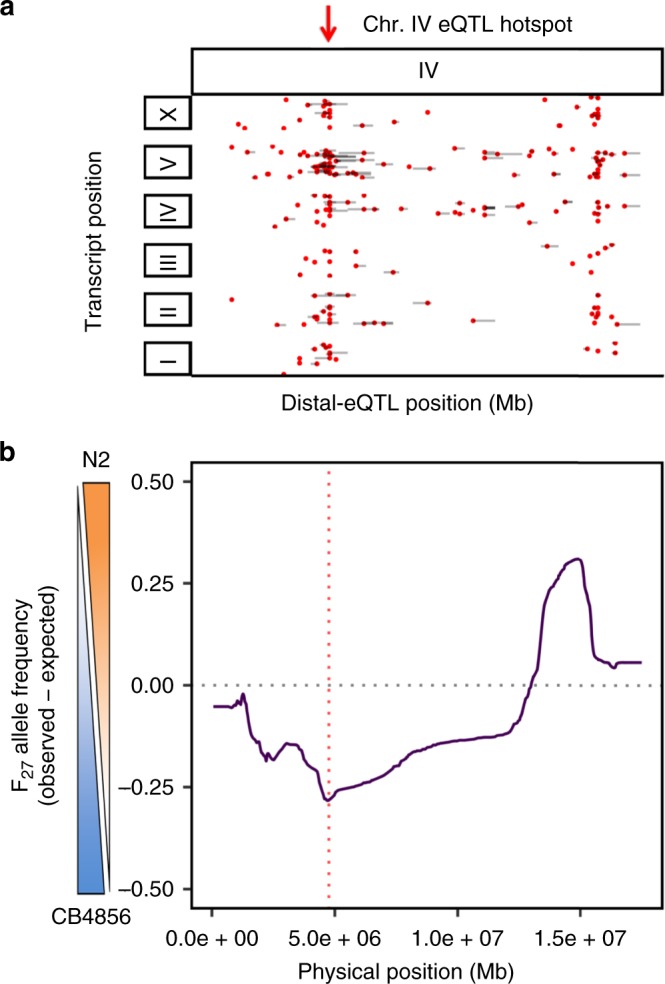
A locus in the left arm of Chr. IV confers a fitness advantage to CB4856. **a** Position of distant expression quantitative trait loci (eQTLs) (red circles) mapping to Chr. IV in a N2 × CB4856 cross^40^; for simplicity, local eQTLs are not shown. Gray bars represent 1 − LOD (logarithm of the odds) drop confidence intervals. Position of the eQTL hotspot on the left end of Chr. IV is highlighted with a red arrow. This hotspot associates with the expression of 47 genes located across the *C. elegans* genome. **b** Allele frequencies of a *C. elegans* eXtreme QTL segregant pool expanded for 27 generations. Values correspond to the difference between the observed and the expected (0.5) in the absence of any selective force. The peak of the fitness locus on the left arm is highlighted with a red dashed vertical line

Our data also revealed loci with antagonistic effects on fitness residing on the same chromosome. The first generations of our segregant pools showed weak but consistent selection in favor of CB4856 alleles on Chr. IV. However, by generation ten, it became apparent that the right arm of Chr. IV was being selected in favor of N2 (Fig. 4). We hypothesized that this selection pattern could emerge if variants with opposite effect on fitness were in linkage. To further examine this possibility, we propagated a *ce*X-QTL segregant pool for a total of 27 generations to accumulate more recombination events between the two fitness loci. Changes in allele frequencies in these advanced generations strongly suggested the presence of two independent loci on Chr. IV with antagonistic effects on fitness, with the left arm being selected in favor of CB4856 and the right arm in favor of N2 (Supplementary Fig. 10c). Comparing our *ce*X-QTL data with expression QTLs (eQTLs) mapped in a previous study revealed that the fitness locus on the left arm of Chr. IV overlapped with a known eQTL hotspot^40^, suggesting that this eQTL could underlie the fitness locus (Fig. 5a, b).

## Discussion

We have developed a novel method in *C. elegans* to quickly and cost-effectively dissect complex traits using millions of animals in bulk. Our approach can be readily adapted to other selfing nematodes. Furthermore, our bulk selection and genotyping approach will greatly facilitate studies of genetic variation in outcrossing nematodes^41^, where abolishing hermaphroditism is not required and inbreeding depression has hindered the generation of panels of inbred lines^42^. Our method offers many advantages over available mapping approaches. For any trait of interest that is amenable to bulk selection, it dramatically reduces the time and work required for genetic mapping compared to using large panels of RILs and wild isolates^2^. Moreover, it lowers the variance of and provides an internal control for phenotypic assays because all individuals are grown and selected in a homogeneous environment. Lastly, it can be easily expanded to study different genetic backgrounds without the need to construct, maintain, genotype, and phenotype large panels of recombinant lines. Once a *ce*X-QTL segregant pool has been generated, it can be used repeatedly to map different traits, as well as frozen for future use. *ce*X-QTL shares with other pooled mapping approaches the limitations that it is not well suited to estimate QTL effect sizes and detect non-additive effects of multiple loci and that it relies on phenotypes that are amenable to selection in bulk. Thus this method is complementary to other QTL mapping approaches, such as those based on RIAILs^43^ and multiparental experimental evolution panels^44^.

Our results also demonstrate the utility of *ce*X-QTL segregant pools to study selection and experimental evolution in *Caenorhabditis*^45^. In the competitive environment of our segregant pool, we have identified various novel loci influencing fitness. The generation of *ce*X-QTL segregant pools is not restricted to two parental genotypes, thus allowing the study of multiple allelic variants in a single experiment. Currently, the main factor limiting the resolution of *ce*X-QTL and other mapping approaches is the highly nonuniform pattern of genetic recombination in *C. elegans*^46^. Thus we foresee that future implementations of *ce*X-QTL could greatly benefit from either targeting recombination to specific loci using CRISPR/Cas9^47^ or manipulating the endogenous recombination machinery of *C. elegans*^48^.

## Methods

### Worm strains and growth conditions

*C. elegans* was grown using standard methods at 20 °C^49^. Worms were fed with the *Escherichia coli* OP50 on modified nematode growth medium (NGM), containing 1% agar and 0.7% agarose to prevent burrowing of CB4856. A detailed list of all the strains used in this study can be found in Supplementary Data 2. Some strains were provided by the CGC, which is funded by NIH Office of Research Infrastructure Programs (P40 OD010440). To generate the CB4856 *fog-2(q71*) strain, we introgressed the *fog-2* allele into CB4856 by performing nine rounds of backcross and selection for the feminization phenotype using CB4856 males. To confirm the introgression, we sequenced the resulting strain using Illumina short-read sequencing. The CB4856 *fog-2(q71)* strain carried only CB5856 variants with the sole exception of a ~1 Mb region in right arm of Chr. V where the *fog-2(q71)* allele is located (Supplementary Fig. 1).

### Generation of *ce*X-QTL segregant populations

We transferred ~150 N2 *fog-2(q71)* virgin L4 hermaphrodites and ~150 CB4856 *fog-2(q71)* males into a single 5 cm NGM plate. Twenty-four hours later, worms and eggs were washed off from the plate and eggs were collected by hypochlorite treatment. The F_1_ generation was synchronized as L1 by starvation overnight in M9 buffer and seeded in three 15 cm NGM plates. After 3 days, once all the “females” were fully gravid, eggs were once again isolated by hypochlorite treatment and L1s synchronized overnight in M9. We repeated this cycle for multiple generations, seeding 50,000 L1s every generation (~2500 L1s per NGM plate) and freezing the rest of the population for long-term storage. Owing to the short life cycle of *C. elegans*, it only takes ~1 month to propagate the population for ten generations. Typically, we recovered a total of ~500,000 L1s every cycle. One generation before a *ce*X-QTL experiment, we further expanded the segregant pool by seeding >250,000 L1s in NGM plates. This expansion guaranteed the recovery of over a million L1 segregants of the next generation readily available for mapping. If required, tens of millions of segregants can be easily obtained by carrying out two population expansion cycles in ~1 week. Importantly, a single *ce*X-QTL segregant pool can be used for multiple mapping experiments or it can be frozen for long-term storage. We have successfully thawed and propagated glycerol stocks of segregant pools and used them for mapping experiments.

### Generation of variant list for CB4856

To assemble a list of variants between N2 and CB4856, we reanalyzed published Illumina sequencing of CB4856^50^. Reads were aligned to *C. elegans* reference build WBcel235 and variants were called using four different genotyping software: *platypus*^51^*, varscan*^52^, *Freebayes*^53^, and the Genomic Analysis Toolkit (GATK)^54^. We considered only SNVs identified by at least three methods. The entire process was automated using the *bcbio-nextgen* pipeline (ver. 0.9.9) (https://bcbio-nextgen.readthedocs.io/). The analysis identified 227,228 SNVs. These SNVs were used to generate a custom reference for CB4856. We then filtered these SNVs further, by aligning reads from our CB4856 *fog-2(q71)* strain to both the reference N2 and our custom CB4856 genome build and retaining only SNVs in which >90% of the reads supported the N2 allele in both alignments. We further excluded SNVs in the mitochondrial DNA. The final list of SNVs included 110,176 variants.

### Generation and processing of whole-genome sequencing data

Genomic DNA was extracted using the DNeasy Blood & Tissue Kit (Qiagen). Illumina sequencing libraries were prepared using the Nextera DNA Library Prep Kit (Illumina). We followed the standard protocol with the following exception: we performed agarose size selection of the Nextera libraries, extracting a ~500-bp band. Libraries were sequenced on Illumina Miseq, Hiseq 2500, Hiseq 4000, and Hiseq X sequencers (see Supplementary Data 3 for a description of all sequencing runs). Reads were aligned to the WBcel235 genome. Alignment bam files were sorted and filtered of PCR duplicates using *sambamba*^55^. Finally, allele counts in each SNV were calculated using the program *bam-readcount* (https://github.com/genome/bam-readcount).

### Statistical analysis of *ce*X-QTL

We implemented a previously published statistical method developed by Magwene et al^20^. For each SNV, allele counts are used to calculate a *G* statistic. To account for segregation distortion, we used a modified version of the statistic estimated in Magwene et al: $G \approx \frac{{[\left( {1 - q} \right)\left( {n_2 - n_1} \right) + q\left( {n_3 - n_4} \right)]}}{{2Cq(1 - q)}}$, where *n*_1_ and *n*_3_ are counts of allele A and B in the high (or treatment) population, and *n*_2_ and *n*_4_ are counts of allele A and B in the low (or control) population; *C* is the depth of coverage at each SNV, and *q* is the baseline frequency of allele A (estimated using the control/low population). The raw *G* statistic was smoothed using a weighted average approach, where the smoothed *G*’ statistic for SNV *s* is given by $G^{\prime}_s = \sum_{j \ in \ W}k_jG_j$, where *k* is the genetic distance between SNV *j* and *s*, transformed using the tri-cube kernel function $k_j = \frac{{(1 - D_j^3)^3}}{{S_W}}$; *W* is a window around *s*, so that only SNVs within the window are used to calculate the weighted average. We imposed a cutoff of 12.5 cM (*W* = 25 cM), the lower bound of the values suggested by Magwene et al. To calculate *p* values corresponding to values of *G*’, the null distribution of *G*’ was estimated from the data using a robust fit to the log-normal distribution (as implemented by the *robust* R package). We have written an R package that implements the entire statistical pipeline, *xQTLstats*, and it is available on github (https://github.com/eyalbenda/xQTLstats).

### Simulations of *C. elegans* segregant populations

To determine the power of X-QTL in *C. elegans*, we first sought to accurately simulate the process of propagating a segregant population. We developed a simulation framework, *bulkPop*, where each individual is represented by two haplotype vectors. Mating is implemented in a straightforward way as a process whereby the haplotypes in each parent recombine, followed by random independent segregation of the recombined haplotypes to progeny. Reflecting the recombination rates in *C. elegans*, the probability of each chromosome to undergo a single recombination event was 0.5, and the location of the recombination was determined using a genetic map based on a recombinant inbred line panel between CB4856 and N2^46^. To simulate loci conferring a fitness advantage (“fitness loci”), we randomly select a subset of individuals from the population to produce progeny, and probability of an individual to be chosen to mate was weighted by the genotype in fitness loci. Lethality due to the *peel-1/zeel-1* element is a result of an interaction between the parental and the zygotic genotype, and this interaction was directly simulated to accurately predict the segregation distortion due to the element. Our framework can easily be extended to crosses in other strains or organisms. It is implemented as an R package, *bulkPop* and is available on github (https://github.com/eyalbenda/bulkpop).

### Simulating the power of *ce*X-QTL

We used *bulkPop* to propagate a large population for ten non-overlapping generations. The starting F1 population was 1000 worms, and the population was capped at 50,000 worms, with each mated female generating 10 progeny. After 10 generations, the population was expanded to 1 million worms 100 times, generating a large 100 million “pool” of segregants to use for simulations. To determine the effect of fitness loci on the power of *ce*X-QTL, fitness was modeled as affecting the probability of a male to participate in mating. All loci were modeled as driven by a single factor, and the strength of selection was chosen such that the segregation distortion in the simulated population was similar to the observed distortion in the X-QTL population across generations.

On the large populations, a *ce*X-QTL drug selection experiment (Fig. 1a) was simulated directly by selecting a 5% survivor population, with a random subset of the large population selected as control. To select 5% survivors in a way that guaranteed that loci had a specified effect size (modeled as the variance explained by the locus *V*_e_), the following procedure was used:

- the genotype of each individual in the causal locus was encoded as *g* = {0,1,2}.
- For each individual, a random displacement factor *d* was simulated for each individual from a normal distribution, with *μ* = 0, and $\sigma ^2 = \frac{{1 - V_{\mathrm{e}}}}{{V_{\mathrm{e}} \times V_{\mathrm{l}}}}$, where *V*_l_ is the variance of the vector of genotypes *g* in the causal locus.
- The final score of each individual was *S* = *g* + *d*

On that score, a cutoff of 5% was imposed to select the survivors of drug selection. Allele counts were simulated based on the allele frequencies in each population using the binomial distribution and used as input for *xQTLstats*.

### Selection for Abamectin resistance

We propagated a N2 × CB4856 segregant pool for 12 generations in NGM plates. We incubated 4 million F_12_ L1 larvae in 0.2 µg/mL Abamectin (Sigma-Aldrich) in M9. Abamectin was freshly dissolved from a 10 mg/mL stock in DMSO kept at −20 °C. L1 larvae were incubated in Abamectin for 1 min and washed three times with 15 mL of M9 buffer. After the washing steps, L1 larvae were seeded on OP50 NGM plates at a density of ~200,000 larvae per plate. Approximately 0.1% of larvae survived this treatment and developed into adult females and males. Surviving adults were washed off from plates and pooled for DNA extraction. As a control, ~5000 F_12_ larvae from the same population were exposed to an equivalent dose of the vector DMSO for 1 min, seeded on OP50 NGM plates, and collected for DNA extraction when the population developed into adults.

To determine the 95% confidence interval for the identified QTL, we simulated a drug selection study using the procedure detailed in the above section. Simulating the population size in our study was computationally unfeasible, so we used a smaller population of 50,000 individuals with 5% surviving the drug treatment. Sequencing was simulated at 100× depth of coverage. The variant with the strongest association with Abamectin resistance in our experiment was taken as the underlying causal variant in the simulation, with an effect size corresponding to 15–25% of the phenotypic variance. We chose that range since the QTL on *V* was estimated to explain up to 25% of phenotypic variance in the previous mapping study^18^. In total, we carried out 1300 iterations. On each iteration, we identified the position of the top associated variant. Finally, the confidence interval was estimated as the interval that encompassed 95% of the top variants across all iterations.

### Selection for H_2_O_2_ resistance

We propagated a N2 × CB4856 segregant pool for 10 generations in NGM plates. We exposed 1.5 million F_10_ L1 larvae to 0.5 M H_2_0_2_ (Sigma-Aldrich) in M9 buffer for 4 h. Approximately 0.1% of larvae survived this treatment and developed into adult females and males. Larvae were washed three times with 15 mL of M9 buffer. After the washing steps, L1 larvae were seeded on OP50 NGM plates at a density of ~200,000 larvae per plate. Approximately 0.1% of larvae survived this treatment and developed into adult females and males. As a control, ~5000 F_12_ larvae from the same population were incubated in M9 for 4 h, seeded on OP50 NGM plates, and collected for DNA extraction when the population developed into adults.

### Selection for hsp-90 reporter expression levels

A single copy^56^ *hsp-90p::GFP::hsp-90* 3’UTR transgene reporter in the N2 genetic background (BCN1082^57^) was introgressed into the CB4856 genetic background by performing six rounds of backcross and selection. We then crossed the N2 and CB4856 *hsp-90p::*GFP reporter lines to their respective *ce*X-QTL parental strains carrying the *fog-2(q71)* mutation. The resultant strains QX2314 (N2 *fog-2(q71)* V; *hsp-90::GFP II*) and QX2307 (CB4856 *fog-2(q71)* V*; hsp-90::GFP* II) were used to generate a *ce*X-QTL segregant population. Fluorescence-based sorting was carried out with a Large Particle Biosorter (Union Biometrica) equipped with a 250-µm Fluidics and Optics Core Assembly (FOCA). Worms were grown on plates to young adult stage (2.5 days after seeding synchronized L1 larvae), washed into 50 mL conical tubes (target concentration 1 worm/µL), and immediately sorted. Worms were anaesthetized by adding Levamisole to 3 mM final concentration.

### RNAi screening

To determine a list of candidates in the deletion on Chr. V, we reanalyzed RNA-seq data from the modEncode project representing gene expression from large synchronized worm populations collected at different developmental stages^24^. Gene expression was quantified from raw sequencing reads using Kallisto^58^. We selected 20 genes that were expressing throughout life for RNAi screening. We used the Ahringer RNAi library (Source Biosciences). We blindly screened RNAi clones targeting 20 genes (Supplementary Data 1) in NGM plates supplemented with Ampicillin (100 µg/mL) and IPTG (1 mM). N2 worms carrying a hsp-90::GFP single copy reporter were scored for increased GFP fluorescence using a stereoscope equipped with a fluorescence lamp.

### Microscopy

Worms were transferred to a 3% Agarose pad and visualized using a Nikon Eclipse 90i microscope equipped with a Photometrics CoolSNAP HQ2 CCD camera.

### Testing candidate variants affecting fitness

We crossed the parental CB4856 *fog-2(q71)* strain to a modified N2 *fog-2(q71)* strain carrying a hypomorphic *peel-1/zeel-1(ttTi12715)* allele carrying a transposon insertion (Chr. I, introgressed from QX1430), a functional *plg-1* allele (Chr. III, introgressed from CB5203), and the WT (CB4856) allele of *npr-1* (Chr. X, introgressed from QX1430). All the alleles were verified in the final strain by PCR or Sanger sequencing.

### Estimation of selection coefficients

We used our *bulkPop* package to simulate the effect of the *peel-1/zeel-1* element on allele frequencies under 20 different values of selection coefficient, which was modeled as the penetrance of the element between 0 (no lethality) and 1 (full lethality). For each value, we simulated 50 populations of 10,000 worms for 20 generations. To estimate the observed selection for the *peel-1/zeel-1* element in the ceX-QTL populations, for each experiment, for each generation, we identified the variant showing the maximum deviation toward the N2 allele. For the experiments carried out with fully functional *peel-1/zeel-1*, that value was averaged across the different experiments. We then identified the best fit to the simulated populations as the value minimizing the sum of the differences between the average of the simulations and the observed across all generations.

### Analysis of the fitness peak in Chr. IV

Microarray genotype and gene expression data for our published expression QTL data^40^ were acquired from the gene expression omnibus. To eliminate discrepancies in gene annotations, probe sequences were realigned to the WBcel235 transcriptome using BWA^59^. Only uniquely mapping probes were used. Expression probes that were present in <2/3 of the sample were removed. The genotype and expression matrices were normalized to have mean zero and variance one. To map eQTLs, we calculated the Pearson correlation between each probe and every genotype. Correlation coefficients were transformed to logarithm of the odds (LOD) scores using $-n\;x\frac{{{\mathrm{ln}}(1 - R^2)}}{{2x\;{\mathrm{ln}}(10)}}$. To assess significance and account for multiple testing, we permuted the sample identities 100 times and calculated the average number of transcripts with an identified eQTL at different LOD scores. We compared these results to the unpermuted LOD scores to estimate the false-discovery rate^60^ and selected a cutoff corresponding to a rate of 5%.

### Reporting summary

Further information on research design is available in the Nature Research Reporting Summary linked to this article.

## Supplementary information

Supplementary Information

Reporting Summary

Description of Additional Supplementary Files

Supplementary Data 1

Supplementary Data 2

Supplementary Data 3

## Data Availability

Raw sequencing data have been deposited in SRA under accession number bioproject PRJNA529922. All *ce*X-QTL figures in this manuscript can be reproduced in Rstudio following the instructions available at https://github.com/eyalbenda/cexQTLview. We have written an R package that implements the entire statistical pipeline, *xQTLstats*, and it is available on github (https://github.com/eyalbenda/xQTLstats).

## Code availability

Custom code and scripts for bulk population simulations and ceX-QTL statistics can be found at https://github.com/eyalbenda/xQTLstats
