# Supplementary material for: Fast genetic mapping of complex traits in C. elegans using millions of individuals in bulk
Source: Nat Commun. 2019 Jun 18;10:2680. doi: 10.1038/s41467-019-10636-9 (PMC6582151; doi:10.1038/s41467-019-10636-9)
Supplement: Supplementary file 1 — Supplementary Information [file 41467_2019_10636_MOESM1_ESM.pdf]

Supplementary Information

Fast genetic mapping of complex traits in C. elegans using millions of individuals in bulk

Burga et al.

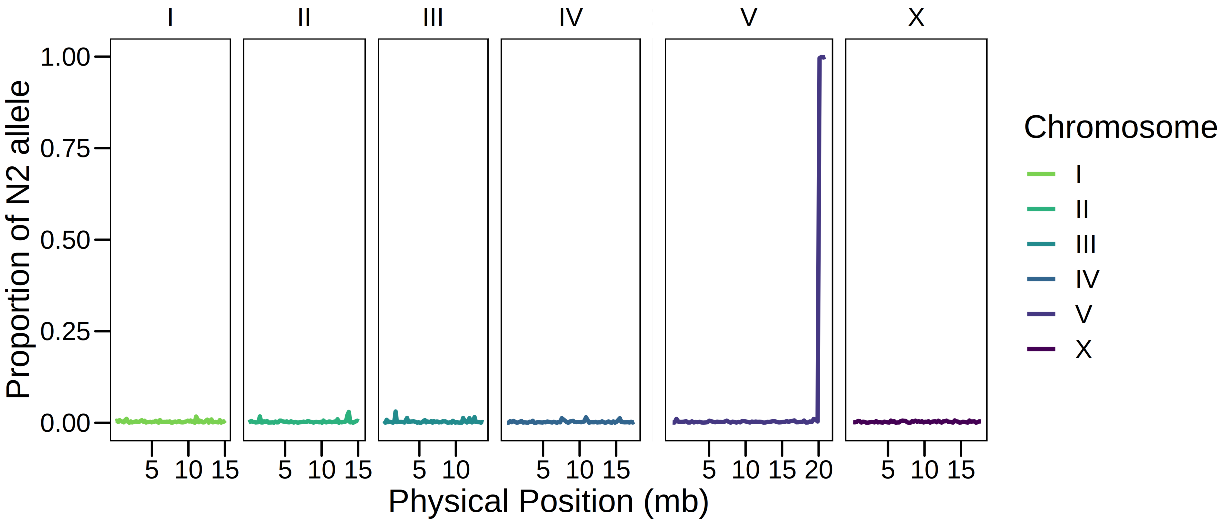

Supplementary Fig. 1. Verification of the CB4856 *fog-2(q71)* introgression strain. Genotyping of the introgression strain was performed using Illumina short read sequencing after 9 backcrosses into CB4856 parental strain. The resulting strain contains a ~1 Mb N2 region in the right end of Chr. V where the *fog-2* gene is located. The introgressed region is not considered for X-QTL mapping.

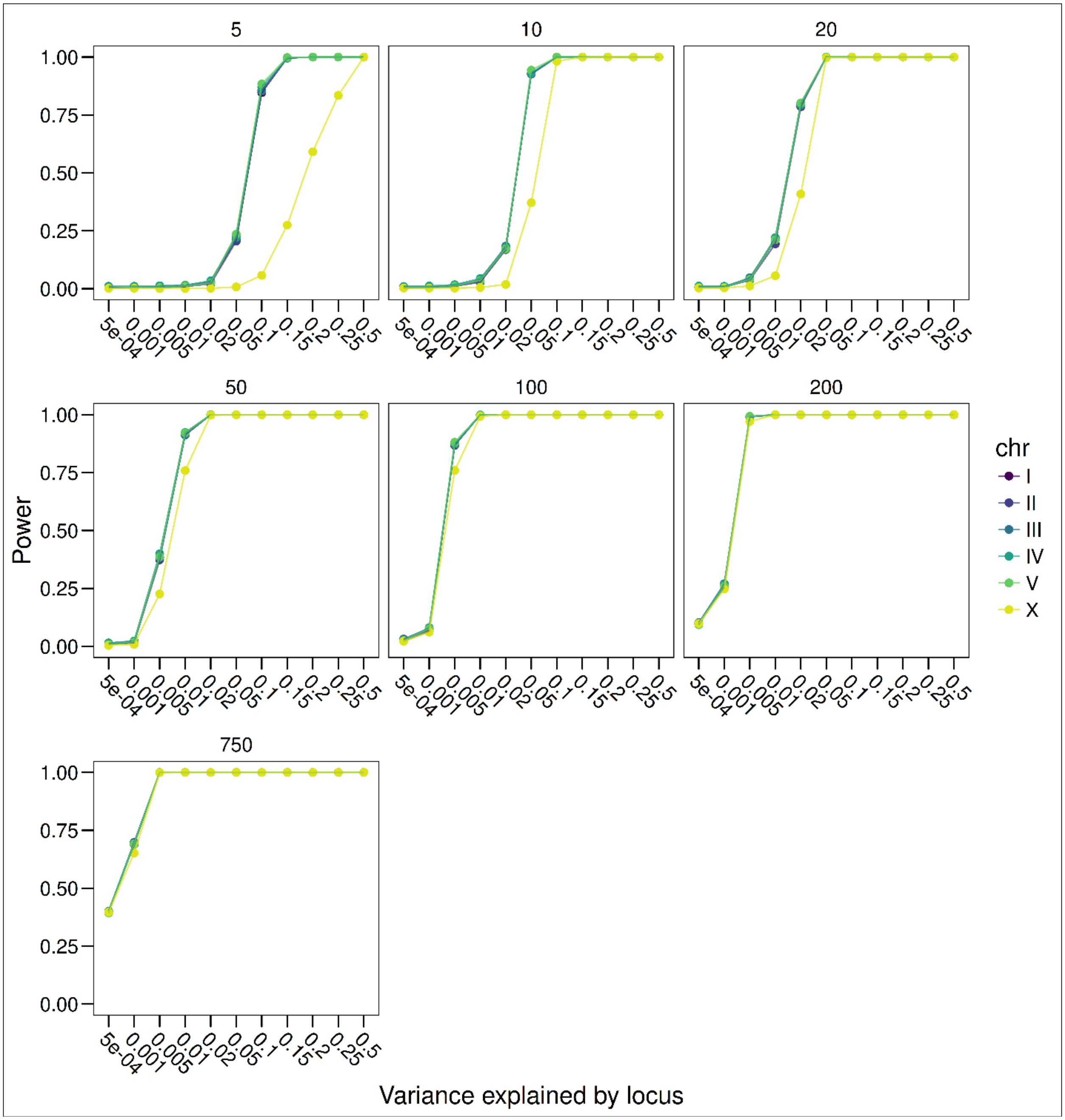

Supplementary Fig. 2. Worm X-QTL is a powerful method for mapping traits across a spectrum of effect sizes. An X-QTL for drug selection (survivors VS. background) was simulated. In each simulation, a population of 50,000 simulated F10 segregants were subjected to a selection with 5% survivors. Loci affecting survival were simulated with effect sizes ranging from 0.05% to 50% of variance explained. Allele counts were simulated from each population with sequencing depth ranging from 5x to 750x. 10 equally distanced loci were simulated across each chromosome. With coverage of 100x (~10GB of sequencing), X-QTL has power to detect loci explaining as little as 0.5% of the phenotypic variance.

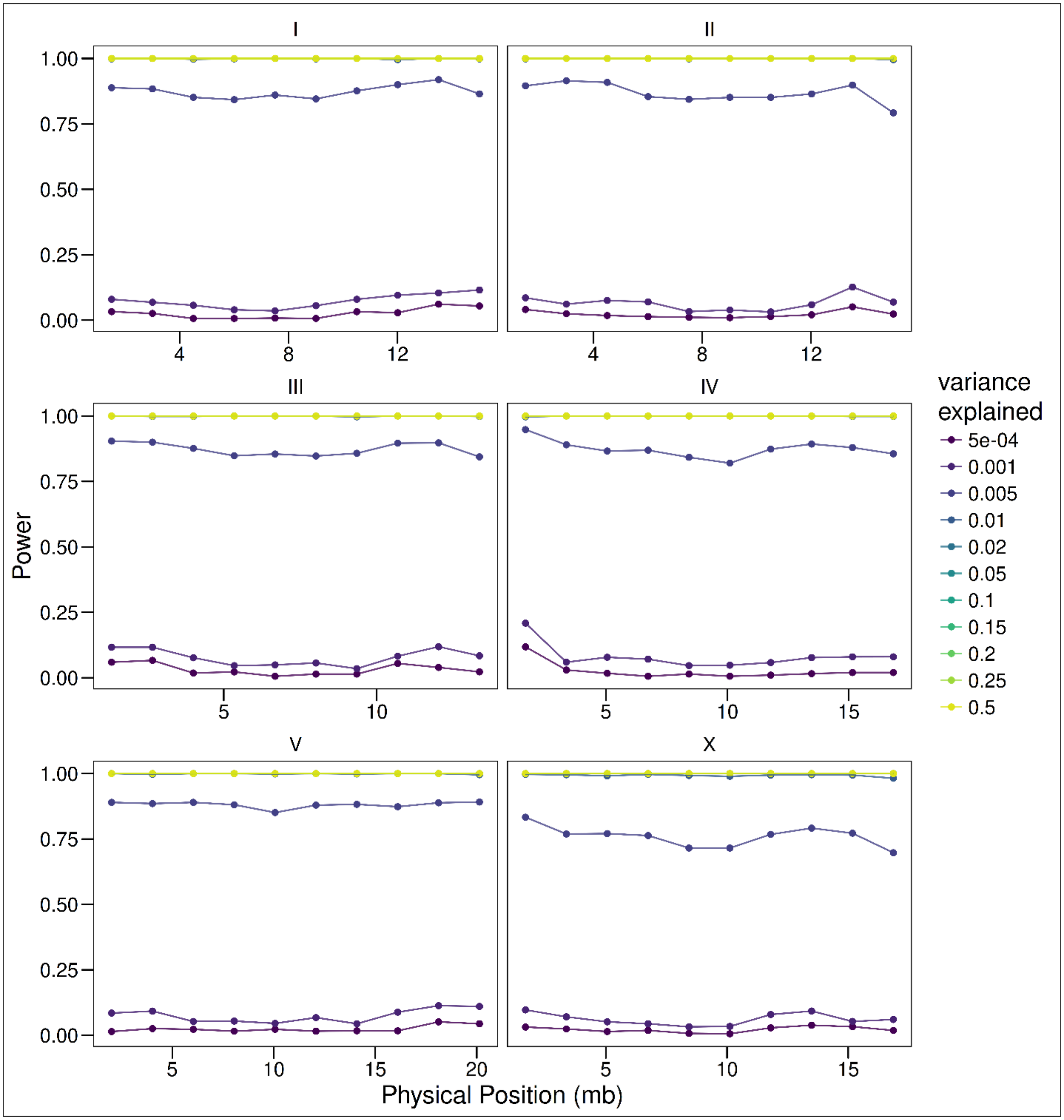

Supplementary Fig. 3. Power of X-QTL across chromosomes. Simulations were carried out as described in Supplementary Fig. 2. 10 loci were simulated at equal distances across each chromosome. With a sequencing depth of 100x (~10 GB of sequencing), X-QTL maintains the power to detect loci above 0.5% variance explained across the *C. elegans* genome. All effect sizes above 0.02 are always detectable in our simulations.

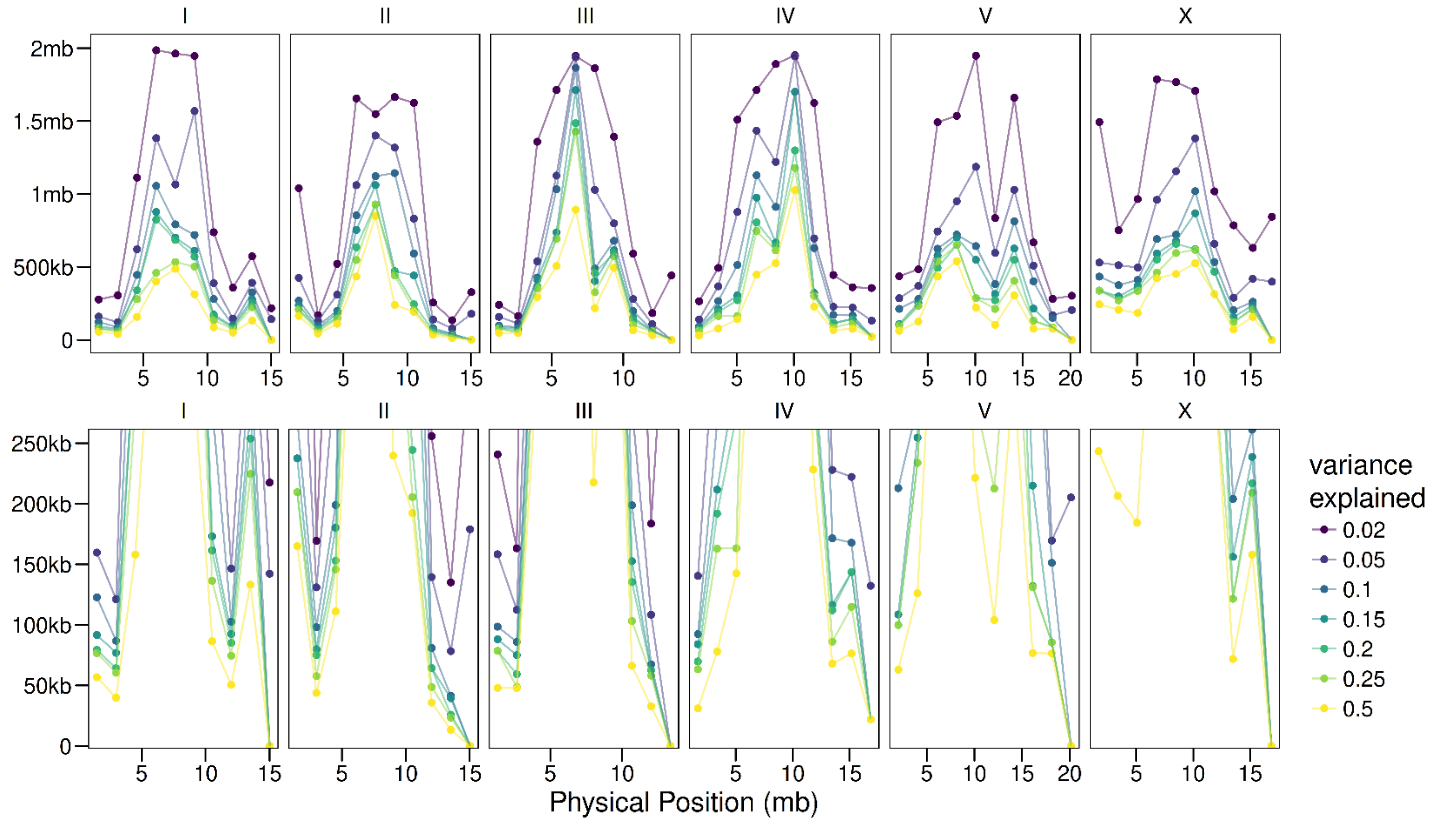

Supplementary Fig. 4. Resolution of X-QTL varies across the chromosome. Simulations were carried out as described in Supplementary Fig. 2, focusing on simulations with a sequencing depth of 100X. For each simulation, the peak position of the mapped X-QTL peak was taken, for a total of 1000 repeats per position. The resolution was defined as the range encompassing 95% of the peaks. As expected, the resolution varies widely across *C. elegans* chromosomes, due to the highly uneven recombination rates. Bottom panel focuses on a range from 50kb-250kb, while top panel shows the entire range.

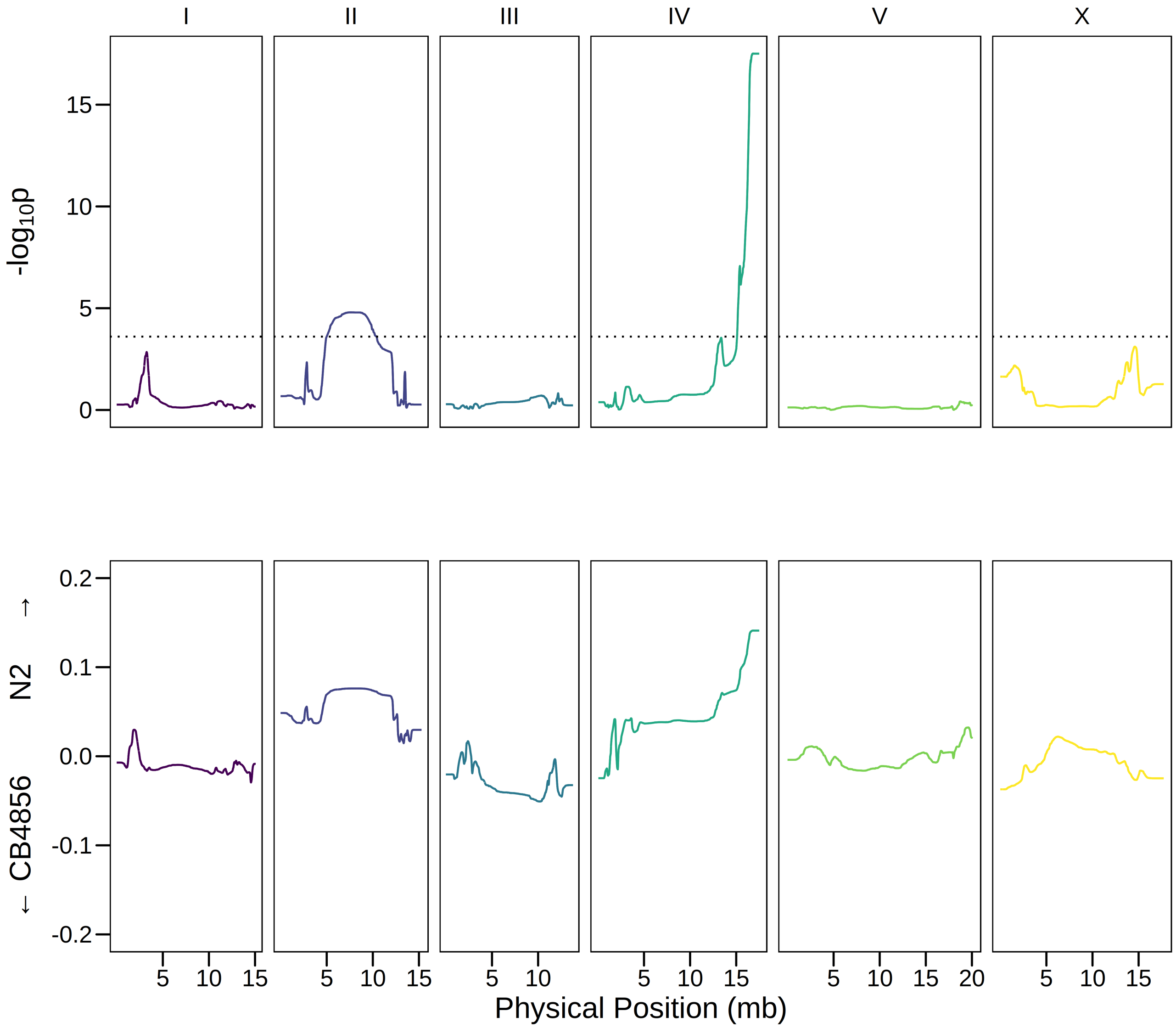

Supplementary Fig. 5. X-QTL mapping identifies loci conferring resistance to $H_2 0_2$. Two significant loci were identified, on chromosomes II ($p=1.60x10^{-5}$) and IV ($p=3.07x10^{-18}$)

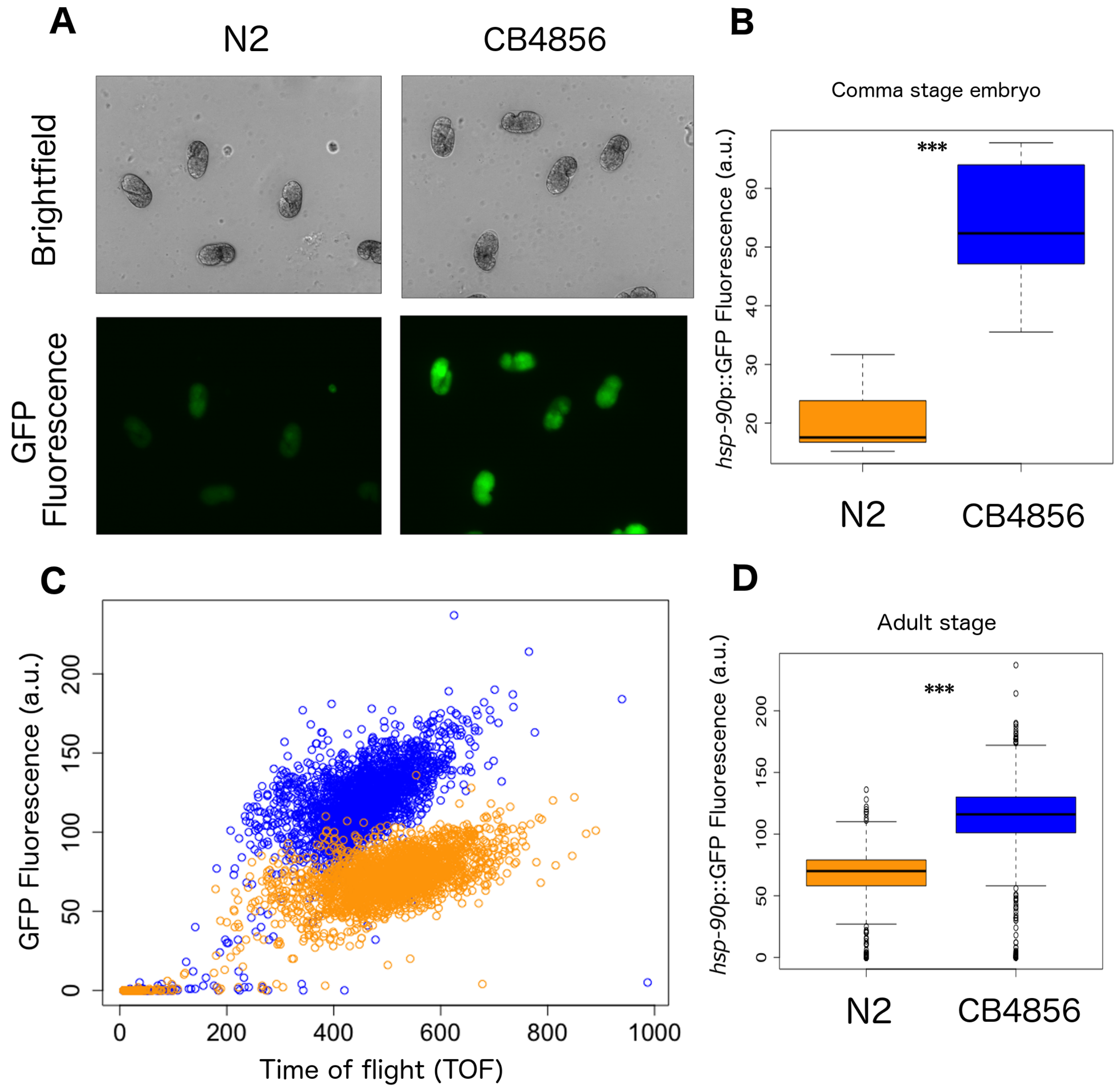

Supplementary Fig. 6. *Hsp-90*::GFP reporter expression levels are upregulated in the CB4856 background. a, Representative images of *hsp-90::GFP* comma stage embryos in both N2 and CB4856 background. **b,** Quantification of GFP fluorescence. *hsp-90::GFP* is upregulated in CB4856 (2.6 fold upregulation; $p=1.0x10^{-4}$, n=7 embryos). **c**, Quantification of GFP fluorescence in adult N2 and CB4856 *hsp-90::GFP* expressing worms using a COPAS Biosorter. **d,** *hsp-90::GFP* is upregulated in CB4856 (1.7 fold upregulation; $p=2.2x10^{-6}$, n= ~2,500 individuals)

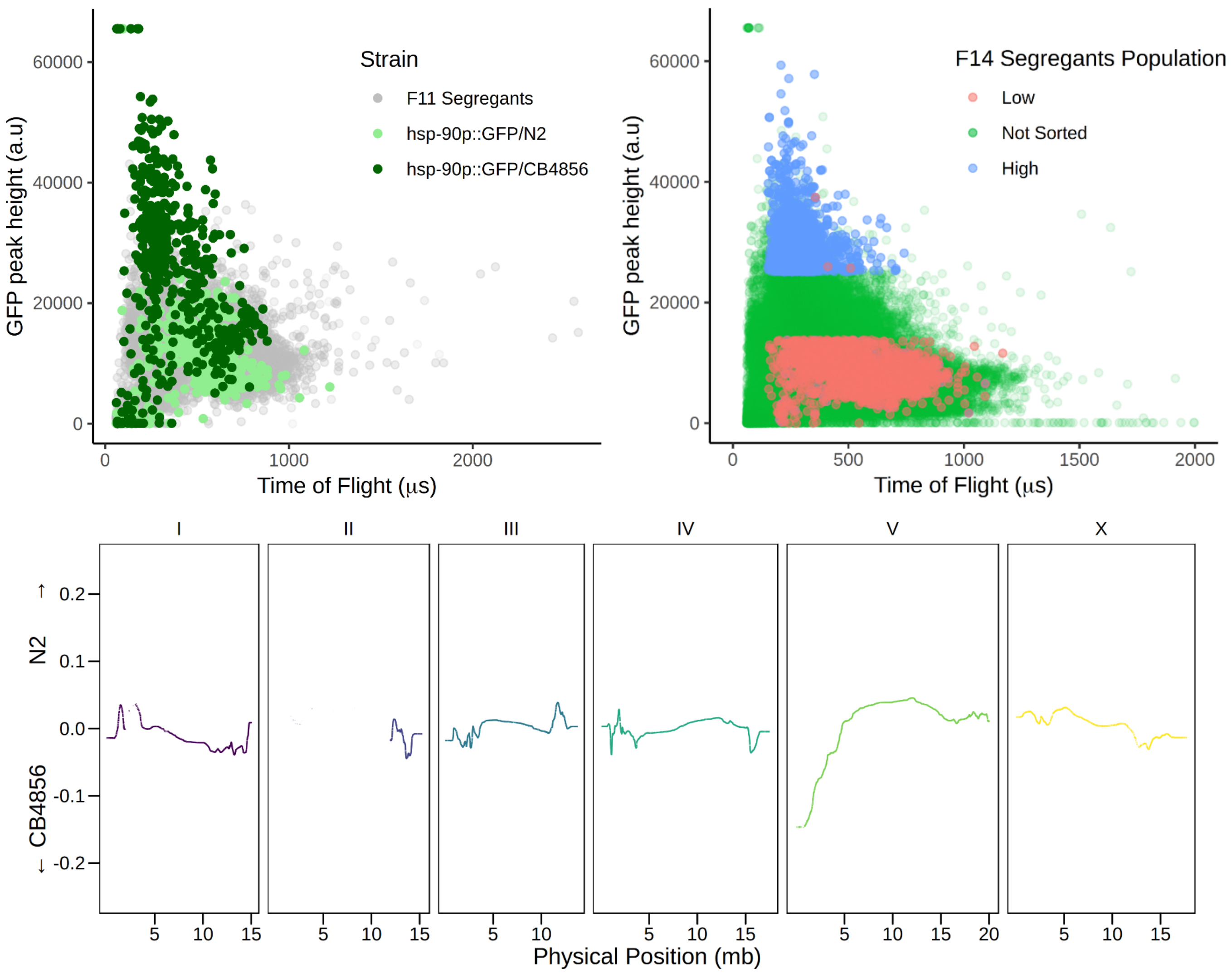

Supplementary Fig. 7. X-QTL mapping for *hsp-90* expression levels. **a,** Sorting of the parental X-QTL strains and F11 segregants (Left), and F_{14} N2 x CB4856 segregant pool carrying the *hsp-90*::GFP transcriptional reporter (Right). **b,** Differences in allele frequencies between the ‘High’ and ‘Low’ GFP fluorescence sorted pools. The ‘High’ GFP expression pool is enriched for CB4856 at the QTL locus in Chr. V.

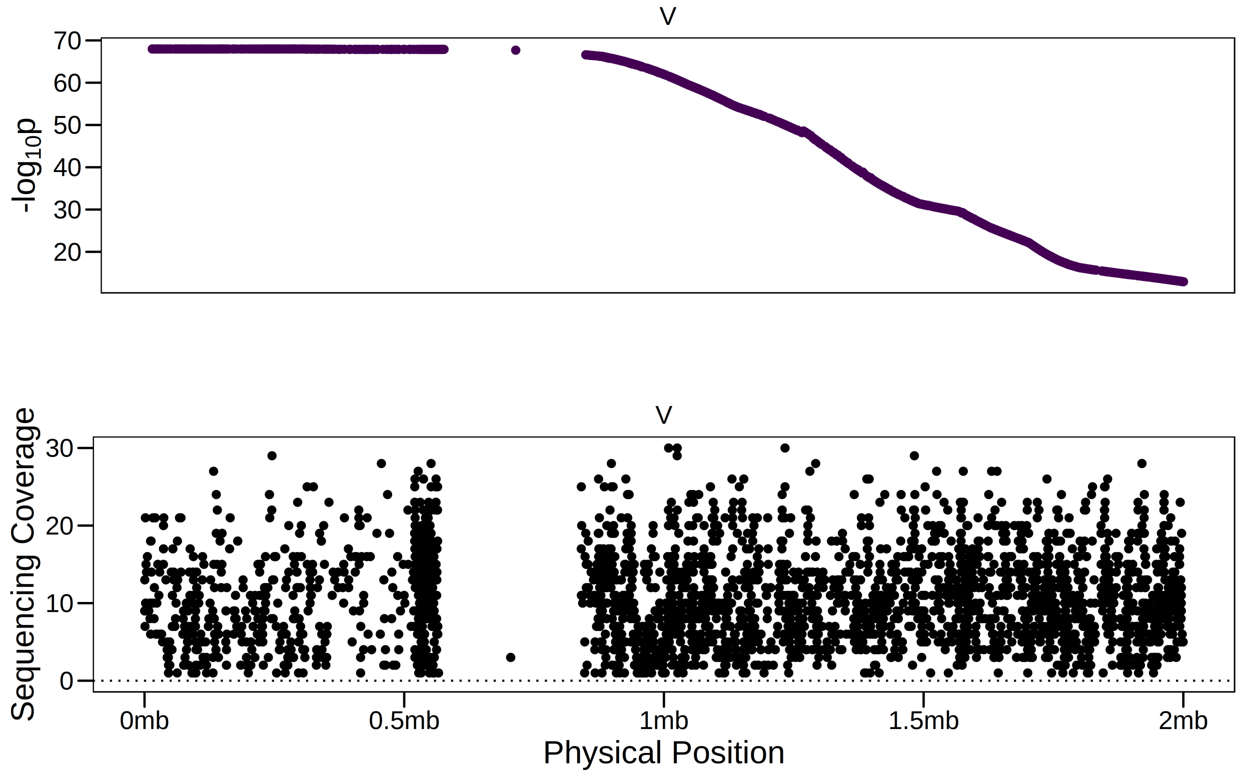

Supplementary Fig. 8. The CB4856 *fog-2(q71) hsp-90*::GFP strain contains a large de novo deletion. (Top) *p-value* for association with GFP signal on the left end of Chr. V. SNVs that are fixed in the population (90% or above) are removed from the analysis. (Bottom) The coverage at SNVs in a strain showing upregulated GFP expression reveals a large region that is depleted, indicating a deletion.

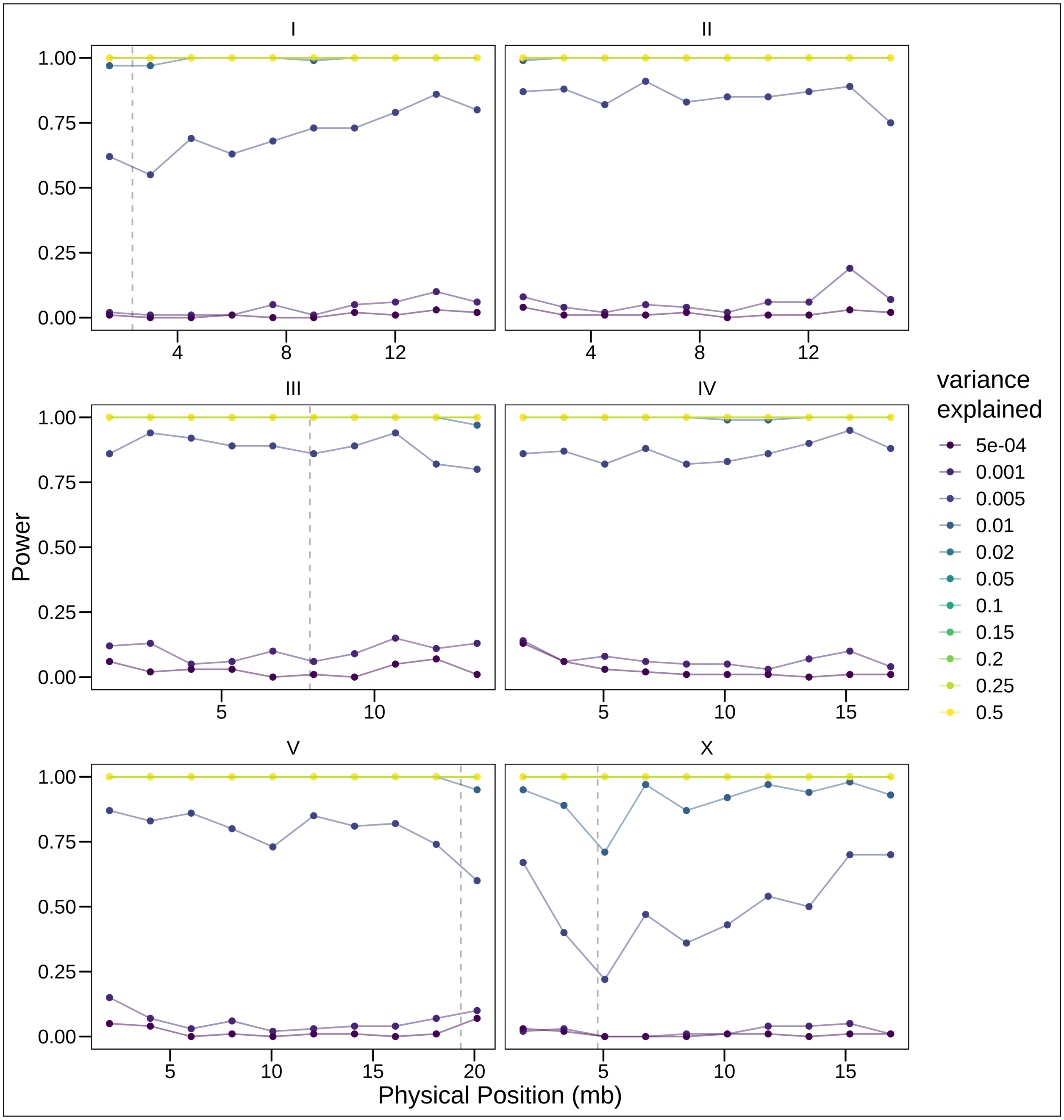

Supplementary Fig. 9. Baseline changes in allele frequencies due to large fitness effects do not prevent X-QTL mapping. Simulations were carried out as described in Supplementary Figure S2, except that we simulated the effect of *peel-1/zeel-1* on Chr I, as well as three loci conferring a fitness advantage on chromosomes III, V, and X as observed in our data (dashed lines). In our simulations, only 20% of the males participate in mating, and we simulated fitness affecting loci as modifiers of the probability of a male to be selected for mating. The specific parameters for each locus were determined by identifying those that resulted in selection strength similar to observed effects. All effect sizes above 0.02 are always detectable in our simulations.

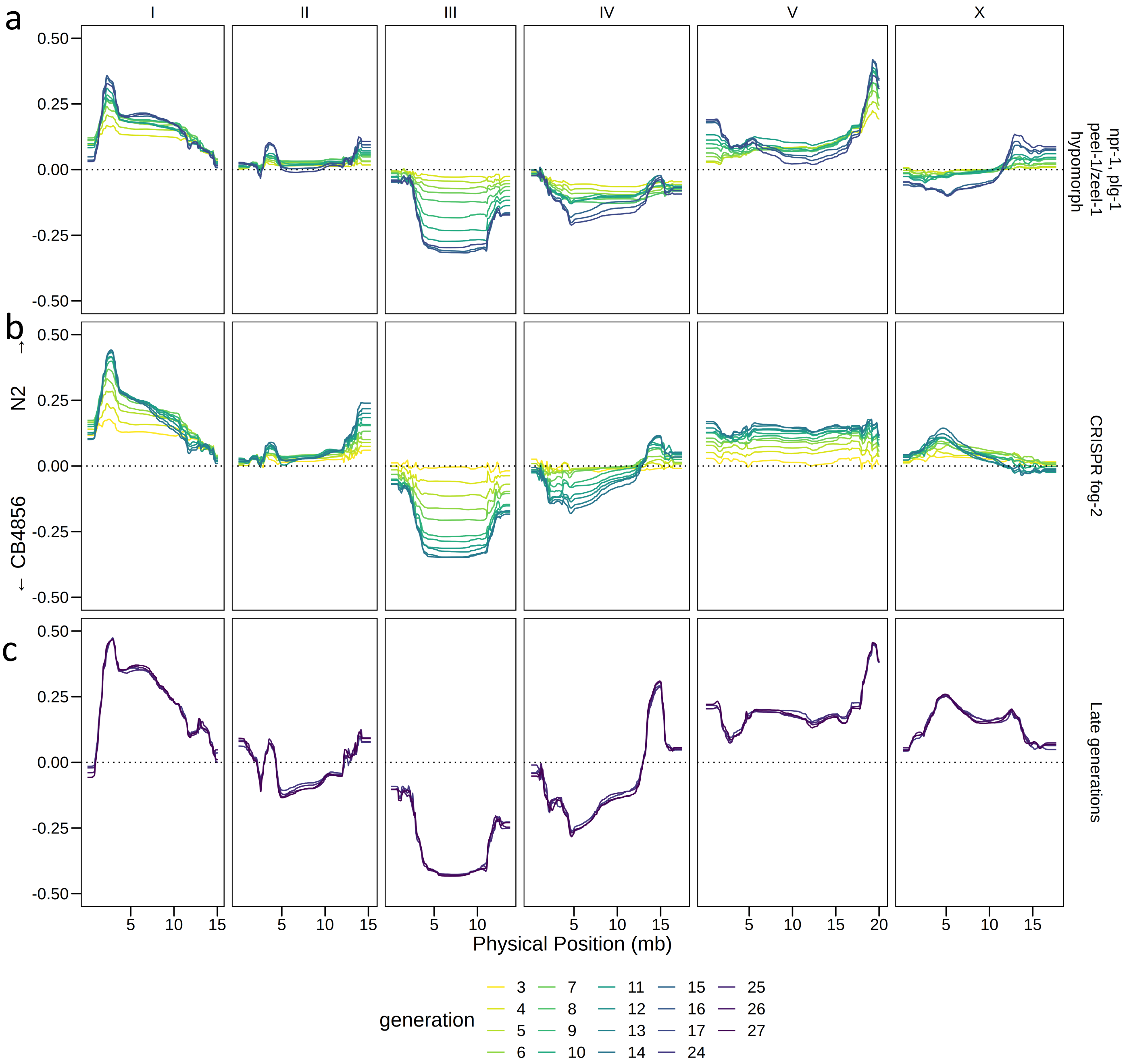

Supplementary Fig. 10. **Additional X-QTL segregant pools**. **a,** We crossed the parental CB4856 *fog-2(q71)* strain to a modified N2 *fog-2(q71)* strain carrying a hypomorphic *peel-1/zeel-1(ttTi12715)* allele (Chr. I, introgressed from QX1430), a functional *plg-1* allele (Chr. III*,* introgressed from CB5203) and the WT allele of *npr-1.* Drive towards N2 in the *peel-1/zeel-1* locus is weaker and selection towards N2 *npr-1* is completely abolished. No changes were observed in Chr. III indicating that variation in *plg-1* does not underlie the selection in favor of CB4856 alleles (compare (A) to Supplementary Figure 10). **b,** Dynamics of an X-QTL segregant pool generated by

replacing the "introgression CB4856 *fog-2(q71)* parental strain" with a CB4856 *fog-2* strain generated by CRISPR/Cas9 targeted allele replacement. Most large fitness peaks are reproduced (compare (B) to Supplementary Figure 10) with the exception of a locus linked to fog-2 (right end of Chr. V) and a secondary peak in Chr X. **c,** allele frequencies of advanced X-QTL generations (F_{24}-F_{27}). Two loci of antagonizing effects on fitness are identified on Chr. IV selecting for CB4856 (left arm of the chromosome) and N2 (right arm).
