# Supplementary material for: Fast genetic mapping of complex traits in C. elegans using millions of individuals in bulk
Source: Nat Commun. 2019 Jun 18;10:2680. doi: 10.1038/s41467-019-10636-9 (PMC6582151; doi:10.1038/s41467-019-10636-9)
Supplement: Supplementary file 2 — Reporting Summary [file 41467_2019_10636_MOESM2_ESM.pdf]

nature research

Corresponding author(s): Burga, Ben-David and Kruglyak

Last updated by author(s): May 1, 2019

Reporting Summary

Nature Research wishes to improve the reproducibility of the work that we publish. This form provides structure for consistency and transparency in reporting. For further information on Nature Research policies, see Authors & Referees and the Editorial Policy Checklist.

Statistics

For all statistical analyses, confirm that the following items are present in the figure legend, table legend, main text, or Methods section.

n/a	Confirmed	
☐	☒	The exact sample size (*n*) for each experimental group/condition, given as a discrete number and unit of measurement
☐	☒	A statement on whether measurements were taken from distinct samples or whether the same sample was measured repeatedly
☐	☒	The statistical test(s) used AND whether they are one- or two-sided<br>*Only common tests should be described solely by name; describe more complex techniques in the Methods section.*
☐	☒	A description of all covariates tested
☐	☒	A description of any assumptions or corrections, such as tests of normality and adjustment for multiple comparisons
☐	☒	A full description of the statistical parameters including central tendency (e.g. means) or other basic estimates (e.g. regression coefficient) AND variation (e.g. standard deviation) or associated estimates of uncertainty (e.g. confidence intervals)
☐	☒	For null hypothesis testing, the test statistic (e.g. *F*, *t*, *r*) with confidence intervals, effect sizes, degrees of freedom and *P* value noted<br>*Give P values as exact values whenever suitable.*
☒	☐	For Bayesian analysis, information on the choice of priors and Markov chain Monte Carlo settings
☒	☐	For hierarchical and complex designs, identification of the appropriate level for tests and full reporting of outcomes
☒	☐	Estimates of effect sizes (e.g. Cohen's *d*, Pearson's *r*), indicating how they were calculated

Our web collection on statistics for biologists contains articles on many of the points above.

Software and code

Policy information about availability of computer code

Data collection	N/A
Data analysis	Custom software can be found at github.com/eyalbenda/bulkpop.<br>All ceX-QTL figures in this manuscript can be reproduced in Rstudio following the instructions in https://github.com/eyalbenda/cexQTLview.<br><br>R, platypus, varscan, Freebayes, GATK, bcbio-nextgen pipeline (ver. 0.9.9), sambamba, bam-readcount (https://github.com/genome/bam-readcount).

For manuscripts utilizing custom algorithms or software that are central to the research but not yet described in published literature, software must be made available to editors/reviewers. We strongly encourage code deposition in a community repository (e.g. GitHub). See the Nature Research guidelines for submitting code & software for further information.

Data

Policy information about availability of data

All manuscripts must include a data availability statement. This statement should provide the following information, where applicable:

- Accession codes, unique identifiers, or web links for publicly available datasets
- A list of figures that have associated raw data
- A description of any restrictions on data availability

Raw sequencing data has been deposited in SRA under accession number bioproject PRJNA529922. All ceX-QTL figures in this manuscript can be reproduced in Rstudio following the instructions in https://github.com/eyalbenda/cexQTLview. We have written an R package that implements the entire statistical pipeline, xQTLstats, and it is available on github (github.com/eyalbenda/xQTLstats).

Field-specific reporting

Please select the one below that is the best fit for your research. If you are not sure, read the appropriate sections before making your selection.

☒ Life sciences ☐ Behavioural & social sciences ☐ Ecological, evolutionary & environmental sciences

For a reference copy of the document with all sections, see nature.com/documents/nr-reporting-summary-flat.pdf

Life sciences study design

All studies must disclose on these points even when the disclosure is negative.

Sample size	Sample size of ceX-QTL mapping population was determined using power simulations in R
Data exclusions	No data was excluded
Replication	We performed biological replicates of our mapping experiments
Randomization	N/A
Blinding	N/A

Reporting for specific materials, systems and methods

We require information from authors about some types of materials, experimental systems and methods used in many studies. Here, indicate whether each material, system or method listed is relevant to your study. If you are not sure if a list item applies to your research, read the appropriate section before selecting a response.

Materials & experimental systems

n/a	Involved in the study	
☒	☐	Antibodies
☒	☐	Eukaryotic cell lines
☒	☐	Palaeontology
☐	☒	Animals and other organisms
☒	☐	Human research participants
☒	☐	Clinical data

Methods

n/a	Involved in the study	
☒	☐	ChIP-seq
☐	☒	Flow cytometry
☒	☐	MRI-based neuroimaging

Animals and other organisms

Policy information about studies involving animals; ARRIVE guidelines recommended for reporting animal research

Laboratory animals	C. elegans N2 strain and isolate CB4856
Wild animals	N/A
Field-collected samples	N/A
Ethics oversight	No special protocols are required for work with nematodes

Note that full information on the approval of the study protocol must also be provided in the manuscript.

Flow Cytometry

Plots

Confirm that:

☒ The axis labels state the marker and fluorochrome used (e.g. CD4-FITC).

☒ The axis scales are clearly visible. Include numbers along axes only for bottom left plot of group (a 'group' is an analysis of identical markers).

☐ All plots are contour plots with outliers or pseudocolor plots.

☐ A numerical value for number of cells or percentage (with statistics) is provided.

Methodology

Sample preparation	C. elegans strains carrying a fluorescent transgenic reporter
Instrument	Union Biometrica large-particle Biosorter
Software	Standard software provided by Union Biometrica
Cell population abundance	N/A
Gating strategy	All sorted worms were fluorescent, high and low populations were defined as the two tails of the distribution and approximately 2000 individuals were collected for each group. The sorted population was synchronized by L1 starvation.

☐ Tick this box to confirm that a figure exemplifying the gating strategy is provided in the Supplementary Information.
