# Supplementary material for: Fast genetic mapping of complex traits in C. elegans using millions of individuals in bulk
Source: Nat Commun. 2019 Jun 18;10:2680. doi: 10.1038/s41467-019-10636-9 (PMC6582151; doi:10.1038/s41467-019-10636-9)
Supplement: Supplementary file 3 — Description of Additional Supplementary Files [file 41467_2019_10636_MOESM3_ESM.pdf]

Description of Additional Supplementary Files

File Name: **Supplementary Data 1**

Description: Details of RNAi screen to identify the transcriptional regulator of *hsp-90*.

File Name: **Supplementary Data 2**

Description: *C. elegans* strains used in this study

File Name: **Supplementary Data 3**.

Description: Illumina short-read sequencing runs
